# Exploring the mechanisms between socio-economic status and health: Mediating roles of health-related behaviors before and during COVID-19

**DOI:** 10.1371/journal.pone.0288297

**Published:** 2024-01-11

**Authors:** Soowon Park

**Affiliations:** Division of Teacher Education, College of General Education for Truth, Sincerity and Love, Kyonggi University, Yeongtong-gu, Suwon-si, Gyeonggi-do, Republic of Korea; Jashore University of Science and Technology, BANGLADESH

## Abstract

The relationship between individuals’ socioeconomic characteristics and their health outcomes is widely acknowledged. However, the specific mechanisms through which these factors are interconnected have not been studied sufficiently. The current study investigated the association among socio-economic status (education of parents, economic status of family) and perceived health mediated by physical activity and sedentary time (purpose for study or not), before and during Covid-19. Three cross-sectional and population-based representative surveys from 2019, 2020, and 2021 were utilized. Overall, 167,099 Korean adolescents (57,303 in 2019, 54,948 in 2020, 54,848 in 2021) participated. A multi-group structural equation model showed that socio-economic status was associated with perceived health through physical activity and sedentary behaviors. Both measures of socio-economic status were positively associated with sedentary time for study purposes, but negatively associated with purposes other than study. Higher education of parents negatively predicted physical activity, whereas higher economic status of family positively predicted physical activity. The impact of socio-economic status on sedentary time tended to increase after the pandemic. Covid-19 significantly affected adolescents’ health and health-related behaviors. Comprehensive policies considering adolescents’ socio-economic status and their physical activity and sedentary time would mitigate the health effects of the pandemic.

## Introduction

The outbreak of Covid-19 in December 2019 in Korea, which resulted in closure of schools, cancelation of public activities, and the imposition of social distancing, significantly affected adolescents. Studies have reported that health-related behaviors such as lack of physical activity or sedentary lifestyles worsened [1–3]. Limited physical activity and excessive sedentary time lead to health problems in adolescents [4]. In addition, health-related behaviors in adolescents are more likely to persist into adulthood as they become a habit and have enormous lifelong effects. Therefore, understanding the influencing factors and their association with health are crucial for promoting adequate health-related behaviors in adolescents and drafting effective public health policies. Specifically, studying the effects of socio-economic determinants would provide an insight to reduce health inequality.

However, it is unclear how Covid-19 affects the associations between socio-economic status and health-related characteristics (i.e., health-related behaviors, perceived health). Following Covid-19, schools or communities were closed and public activities were cancelled, and adolescents were required to remain at home. With staying time at home increasing, it is reasonable to assume that this quarantine situation would impact adolescents’ behaviors and health. Certain factors such as socio-economic status of family are considered to have a critical impact on adolescents’ physical activity and sedentary time during Covid-19 compared with factors including individual’s biological characteristics (age, gender), psychological characteristics (mental health, cognition, motivation, behavior), or Covid-19 policies [2]. A comprehensive understanding of how socio-economic characteristic of family are associated with adolescents’ health before and during Covid-19 is needed.

Fundamental Cause Theory (FCT) posits that social economical differences creates inequalities in access to resources that are related to health, and these inequalities persist over time and across different diseases and health outcomes [5]. FCT in attempt to explain why despite significant improvements in medical technology and healthcare, health inequalities persist in society. The theory draws on the concept of a fundamental cause, which refers to a set of resources that can be used to treat multiple health conditions. Examples of fundamental causes include education and income. One of the key mechanisms or resources to help explain health inequality of socio-economic differences is health related behaviors [6]. Individuals with higher socio-economic status have greater access to resources that promote healthy behaviors, such as exercise facilities, fresh food, and healthcare. On the other hand, those with lower socio-economic status may be more likely to engage in unhealthy behaviors, such as smoking and sedentary behavior [7].

Substantial studies have examined the relationship between socio-economic characteristic and health-related behaviors, but the results are inconsistent [8, 9]. Using over 1.7 million adults’ data, previous study support that the health behavior (smoking, obesity) is a key mechanism between education-health association [10]. However, a systematic review has revealed that 58% of previous articles reported positive associations between socio-economic status and physical activity, while 42% noted that the relationship was null or even negative [11]. This inconsistency may be attributed to the heterogeneity of measures for socio-economic status. Understanding the unique impact of each measure is a possible way to solve the inconsistency.

Parents’ education and family income are considered important proxies for evaluating the socio-economic status of a family. Even though these two dimensions are interrelated, it has been proposed that they have diverse associations with health-related behaviors. Parental income was positively associated with physical activity during Covid-19, but education of parents was not [12]. Mother’s education is not a critical associating factor for physical activity and sedentary time in adolescents [13], but other studies demonstrate that parents’ education and income negatively predict children’s screen time [14]. These inconsistent results may indicate that even though parents’ education and family income were sub-components of socio-economic status, these factors play different roles to predict adolescents’ health-related behaviors. A clear distinction between parents’ education and family income, not collapsing these two different variables as one component, would shed light on the unique contribution of each variable on health and provide valuable information for disentangled inconsistencies in previous studies.

The association between socio-economic status and sedentary behavior varies by domains of sedentary behaviors [8]. In a meta-analysis, differences in domains of sedentary behavior explained 20.2% of heterogeneity among studies in general, which is larger than that of socio-economic status such as education, resources, and occupation (i.e., 6.9%) [8]. Higher socio-economic status is linked with lower TV viewing time but higher non-TV sitting time in adolescents [15]. In addition, distinguishing whether or not sedentary time is spent on studying is particularly important in Korean adolescents. Korea is one of the countries showing the highest study time in adolescents. For instance, 53% of students spend more than 50 hours per week on study in school, which is much higher than the average in OECD (Organization for Economic Co-operation and Development) countries (14.9%) [16]. Generally, studying is considered a sedentary behavior, with sedentary time increasing in proportion with longer hours of study. Furthermore, Korean parents tend to encourage children to spend more hours studying than indulging in recreational sedentary time such as TV/smartphone viewing. In addition, higher parental education may indicate that they value learning and education, thus they tend to push their children to study. Taken together, it is plausible that the education of parents would have a critical influence on sedentary time for studying purposes.

The current study aimed to quantify the prevalence and socio-economic status correlates of physical activity, sedentary time, and perceived health in adolescents before (2019) and during Covid-19 (2020, 2021). The different contributions of each measure of socio-economic status (i.e., education of parents, economic status of family) to predict adolescents’ perceived health through health-related behaviors (i.e., physical activities and sedentary time) were investigated using representative data. Domains of sedentary time were categorized into time spent for study and purposes other than study.

## Methods

### Participants

Data were obtained from the 15^th^ (2019), 16^th^ (2020), and 17^th^ (2021) Youth Health Behavior Online Survey (www.kdca.go.kr/yhs), a nationally representative cross-sectional survey of Korean adolescents (middle to high school students) to examine adolescents’ health behaviors and health status. The Korea Disease Control and Prevention Agency (KDCA) has been administering the online survey each year since 2005. The survey is administered from June to October every year. A stratified random sampling was conducted. The population was stratified into 39 strata of local groups. Next, 400 middle schools and high schools each were selected. Subsequently, classes for each grade were randomly chosen in 800 schools. The survey is approved by the government and consent procedure was sanctioned by the institutional review board of the KDCA. This study employed data from a distinct group of adolescents each year, using identical measurements. The data were independently collected in 2019, 2020, and 2021, with distinct participants but the same measurement tool. Written informed consent prior to participation was obtained. The study was approved by the ethics review board of Kyonggi University (IRB No. KGU-20220913-HR-091).

### Theoretical model

**Fig 1:**
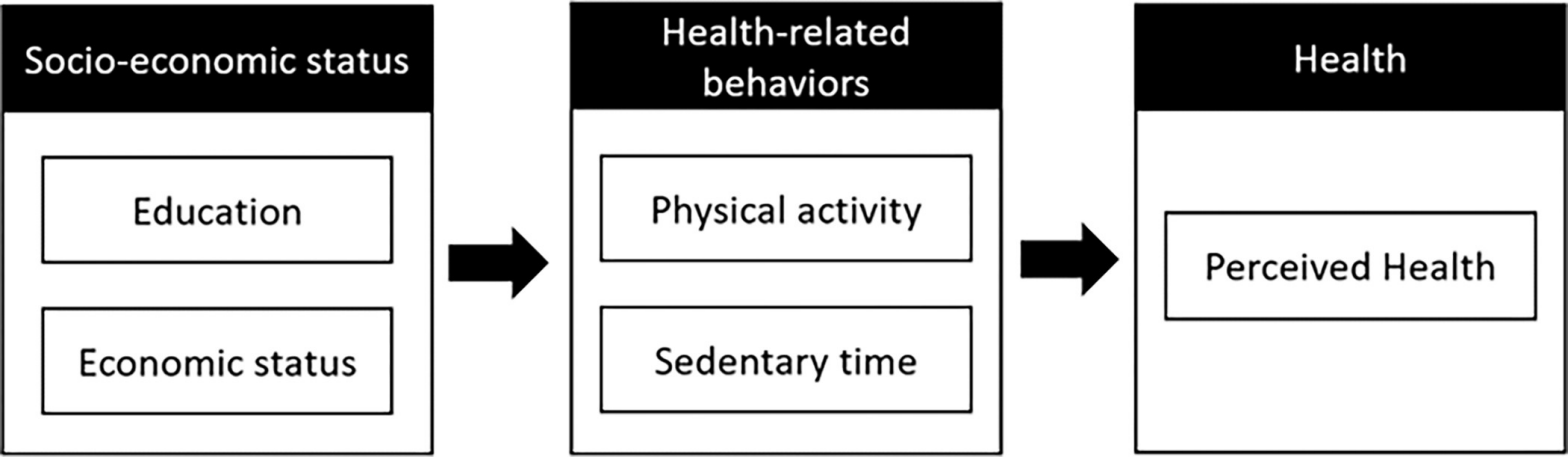
Theoretical model of the current study. The current study adopts the framework of fundamental cause theory to hypothesize that socio-economic status in adolescents, as reflected in parental education and family economic status, is related to health-related behaviors (physical activity and sedentary time). These health-related behaviors, in turn, serve as mediators of the association between socio-economic status and health outcomes.

The purpose of this study is to examine the link between socio-economic factors (i.e., education and economic status) and health, mediated by health-related behaviors (i.e., physical activity and sedentary time) during adolescence. Fig 1 displays the conceptual theoretical model.

### Measures

#### Education of parents

Parental education was measured by the question, ‘What is your educational background?’ The participants were told to answer for their father and mother individually and the responses were recorded using a four-point Likert scale (1 = middle school or less, 2 = high school, 3 = college or more, 4 = I don’t know).

#### Economic status of family

Subjective economic status of the family was measured using the question, ‘What is the economic status of the family?’ on a five-point Likert scale (1 = high, 2 = middle high, 3 = middle, 4 = middle low, 5 = low).

#### Physical activity

Physical activity was measured by two questions regarding vigorous-intensity physical activity and muscle-strengthening exercises. Vigorous-intensity physical activity was measured with the question, ‘Over the past seven days, how many days did you perform high-intensity physical activities (such as jogging, soccer, basketball, taekwondo, mountaineering, fast cycling, fast swimming, and carrying heavy objects) for 20 minutes or more?’ on a six-point Likert scale (1 = None for the past 7 days, 2 = once a week, 3 = 2 times a week, 4 = 3 times a week, 5 = 4 times a week, 6 = 5 times a week or over). Muscle-strengthening exercise was measured by the question, ‘Over the past seven days, how many days did you carry out muscle-strengthening exercises such as push-ups, sit-ups, weightlifting, dumbbells, iron bars, and parallel bars to build muscle strength?’ on a six-point Likert scale (1 = None for the past 7 days, 2 = once a week, 3 = 2 times a week, 4 = 3 times a week, 5 = 4 times a week, 6 = 5 times a week or over). The Cronbach’s alpha scores were .72, .75, and .72 in 2019, 2020, and 2021, respectively.

#### Sedentary time

Sedentary time was assessed by the question, ‘Over the past seven days, how many hours per day on average did you spend sitting?’ The answers were divided into two categories: ‘time spent sitting for study purposes’ and ‘sitting for purposes other than study.’ The sedentary time (hours per day) for weekdays and weekends were measured in an open-ended format.

#### Perceived health

Perceived health was measured by the question, ‘What do you think your health is usually like?’ on a five-point Likert scale (1 = very healthy, 2 = healthy, 3 = normal, 4 = unhealthy, 5 = very unhealthy).

#### Control variables

Age, gender, and academic achievement were used as control variables. Academic achievement was measured by the question, ‘How are your school grades in the last 12 months?’ on a five-point Likert scale (1 = high, 2 = middle high, 3 = middle, 4 = middle low, 5 = low).

### Statistical analysis

The analysis was conducted using SPSS 18.0 (United States, IL). Variables including economic status of family, perceived health, and academic achievement were reverse-coded for high score, indicating the high level of each variable. The answer ‘I don’t know’ in education of parents was treated as missing in the data analysis. The average number of hours of sedentary time for weekdays and weekends were used in the data analysis. Males were coded as 0 and females 1.

To compare the mean difference across years, a one-way analysis of variance (ANOVA) was conducted. Brown–Forsythe Robust one-way ANOVA and Tamhane post-hoc were conducted under heteroscedasticity. To test the association between the variables across years (2019, 2020, 2021), a multi-group structural equation modeling was conducted using AMOS 20.0 (SPSS, USA). Full Information Maximum Likelihood methods were utilized. By examining the fit indices of the models without constraint among the three groups (2019, 2020, 2021), configural invariances were checked. The goodness of model was tested using RMSEA (the Root Mean Square Error of Approximation) and CFI (the Comparative Fit Index). These two indices were relatively stable regardless of sample size and they consider simplicity of model than χ^2^ test. CFI evaluates the overall goodness of fit, while RMSEA focuses on discrepancies between the model-implied and observed covariance matrices. These two measures complement each other and provide information on different aspects of model fit; thus, the current study used these fit indices to examine the goodness of model fit. Models were good fit when the RMSEA was below 0.05 and CFI was over the 0.95 [17]. Invariance among groups was assumed to be satisfied when the differences of the CFI were under 0.01 [18]. Differences between the paths among the groups were tested using critical ratio difference tests.

## Results

### General characteristics of participants

The general characteristics (age, grade, gender, parental education, income of family, academic achievement) of adolescents are presented in Table 1. The mean age was 15 and students in Grade 1 of middle school to Grade 3 of high school participated.

**Table 1:** General characteristics of participants.

	Before Covid-19	During Covid-19	
	2019 (*n* = 57,069)	2020 (*n* = 54,809)	2021 (*n* = 54,712)
Age (Mean ±SD)	14.97±1.78	15.10±1.75	15.09±1.74
School grade			
Grade 1 of middle school	9,738(17.0)	10,005(18.2)	10,016(18.3)
Grade 2 of middle school	9,665(16.9)	9,564(17.4)	10,235(18.7)
Grade 3 of middle school	9,981(17.4)	9,392(17.1)	9,764(17.8)
Grade 4 of high school	9,273(16.2)	8,907(16.2)	8,461(15.4)
Grade 2 of high school	9,044(15.8)	8,907(16.2)	8,647(15.8)
Grade 3 of high school	9,602(16.8)	8,173(14.9)	7,725(14.1)
Gender			
Boys	29,841(52.1)	28,353(51.6)	28,401(51.8)
Girls	27,462(47.9)	26,595(48.4)	26,447(48.2)
Parental education			
Father			
Middle school or lower	615(1.7)	658(1.6)	558(1.4)
High school	8,734(23.8)	9,419(22.9)	8,665(21.7)
College or more	19,980(54.5)	22,638(55.1)	22,958(57.6)
‘I don’t know’	7,303(19.9)	8,381(20.4)	7,680(19.3)
Mother			
Middle school or lower	519(1.4)	486(1.2)	453(1.1)
High school	10,174(27.7)	11,170(26.9)	10,147(25.2)
College or above	19,349(52.6)	22,207(53.5)	22,722(56.5)
‘I don’t know’	6,748(18.3)	7,650(18.4)	6,929(17.2)
Family economic status			
Low	1,299(2.3)	1,275(2.3)	1,112(2.0)
Middle-Low	6,042(10.5)	5,937(10.8)	5,091(9.3)
Middle	27,457(47.9)	26,397(48.0)	27,077(49.4)
Middle-High	16,126(28.1)	15,300(27.8)	15,624(28.5)
High	6,379(11.1)	6,039(11.0)	5,944(10.8)
Academic achievement			
Low	7,647(13.3)	6,736(12.3)	7,084(12.9)
Middle-Low	14,296(24.9)	13,410(24.4)	13,444(24.5)
Middle	17,234(30.1)	16,585(30.2)	16,903(30.8)
Middle-High	12,570(21.9)	12,684(23.1)	12,004(21.9)
High	5,556(9.7)	5,533(10.1)	5,413(9.9)

Note. Prevalence (%) was presented, SD = standard deviation.

### Physical activity, sedentary time, and perceived health before and during Covid-19

Physical activity, sedentary time, and perceived health across the three years are presented in Table 2. Physical activity and sedentary time for study purposes were the lowest immediately after the outbreak of Covid-19 (2020) than before Covid-19 (2019) or later (2021), whereas the sedentary time for purposes other than study was the highest immediately after the Covid-19 outbreak (2020), followed by 2021 and 2019. Total sedentary time gradually increased. Adolescents’ perceived health was lowest in 2021 and it did not differ before Covid-19 (2019) and immediately after the Covid-19 outbreak (2020).

**Table 2:** Trends in physical activity and sedentary time and perceived health.

	2019 (a)	2020 (b)	2021 (c)	*Statistic*	*p*	Post-hoc
Physical activity	2.57(1.52)	2.52(1.58)	2.55(1.53)	16.30	< .001	b < a, b
Sedentary time (hours/day)						
for study purposes	5.75(3.54)	5.11(3.20)	5.65(3.21)	597.84	< .001	b < c < a
for purposes other than study	3.84(2.66)	4.70(3.01)	4.45(2.87)	1320.23	< .001	a < c < b
Total	9.59(4.20)	9.80(4.07)	10.10(3.94)	222.86	< .001	a <b < c
Perceived health	3.89(0.90)	3.89(0.90)	3.77(0.91)	331.63	< .001	c < a, b

Note. Asymptotically F distributed

### Associations among the variables before and during Covid-19

**Fig 2:**
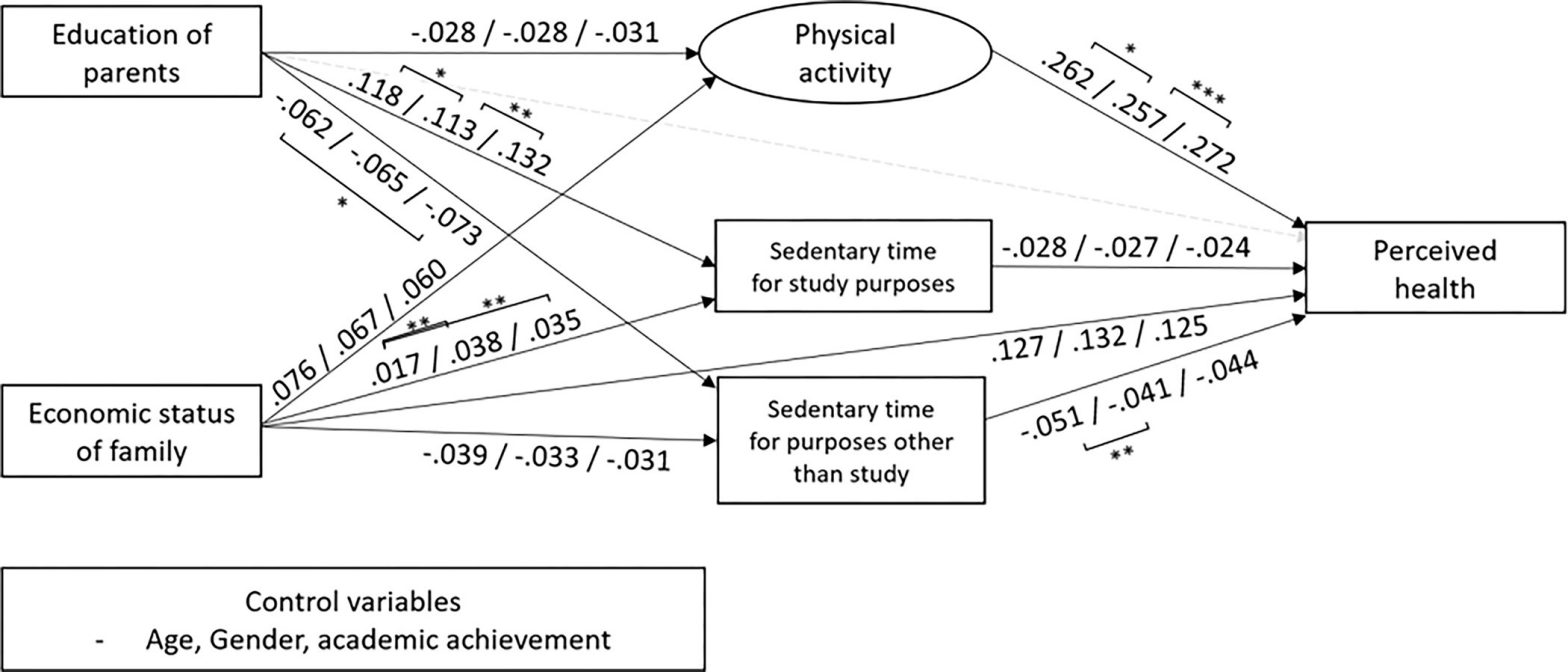
Associations among socio-economic status (parental education, economic status of family), health-related behaviors (physical activities, sedentary time for study purposes, sedentary time for purposes other than study) and perceived health. Significant paths were presented in the solid black lines and standardized regression coefficients of 2019/2020/2021 were presented. In addition, the significant differences in the coefficient across the year were presented as asterisk mark.

The model fits for unconstrained model were *χ*^2^(165) = 4265.749, *χ*^2^/*df* = 142.192, RMSEA = .029, CFI = 976 and those for the measurement invariant model were *χ*^2^(163) = 4690.606, *χ*^2^/*df* = 146.581, RMSEA = .030, CFI = 974. The CFI’s change was under 0.01 between the models, which means that the measurement invariant is satisfied. The measurement invariant model was selected as the final model. The estimates of unstandardized and standardized regression coefficients, standard errors, and critical ratios for all paths are presented in Table 3. Fig 2 shows the significant standardized coefficients among the variables.

**Table 3:** Model estimates in 2019, 2020, and 2021.

						2019				2020		2021		
			b	S.E.	β	C.R.	b	S.E.	β	C.R.	b	S.E.	β	C.R.
Education of parents	→	Physical activity	-0.075	0.017	-0.028	4.482***	-0.080	0.017	-0.028	4.756***	-0.087	0.017	-0.031	5.205***
Economic status of family			0.108	0.007	0.076	15.528***	0.100	0.007	0.067	13.550***	0.089	0.007	0.060	11.925***
Age			-0.085	0.003	-0.118	25.666***	-0.056	0.004	-0.073	15.69***	-0.086	0.003	-0.117	24.719***
Gender			-1.163	0.012	-0.457	96.374***	-1.133	0.013	-0.42	88.21***	-1.088	0.012	-0.424	87.582***
Academic achievement			-0.041	0.005	-0.038	7.908***	-0.025	0.006	-0.022	4.476***	-0.027	0.005	-0.024	4.904***
Education of parents	→	Sedentary time for study purposes	0.879	0.039	0.118	22.348***	0.768	0.035	0.113	22.110***	0.931	0.036	0.132	25.918***
Economic status of family			0.067	0.017	0.017	4.057***	0.135	0.015	0.038	8.790***	0.131	0.016	0.035	8.187***
Age			0.642	0.008	0.322	81.917***	0.504	0.007	0.277	67.960***	0.454	0.007	0.246	60.703***
Gender			1.142	0.027	0.161	41.759***	0.659	0.026	0.103	25.740***	0.898	0.026	0.140	35.005***
Academic achievement			0.658	0.012	0.218	53.53***	0.619	0.012	0.226	53.350***	0.647	0.012	0.235	55.430***
Education of parents	→	Sedentary time for purposes other than study	-0.347	0.032	-0.062	10.768***	-0.417	0.035	-0.065	11.960***	-0.459	0.034	-0.073	13.458***
Economic status of family			-0.115	0.013	-0.039	8.519***	-0.110	0.015	-0.033	7.125***	-0.103	0.015	-0.031	6.777***
Age			-0.098	0.006	-0.065	15.265***	-0.123	0.007	-0.072	16.550***	-0.129	0.007	-0.078	18.189***
Gender			-0.084	0.022	-0.016	3.774***	0.023	0.026	0.004	0.895^*ns*^	-0.148	0.024	-0.026	6.090***
Academic achievement			-0.195	0.010	-0.086	19.462***	-0.288	0.012	-0.112	24.780***	-0.338	0.011	-0.138	30.615***
Education of parents	→	Perceived health	0.009	0.011	0.005	0.836^*ns*^	-0.015	0.01	-0.008	1.439^*ns*^	-0.010	0.011	-0.005	0.942^*ns*^
Economic status of family			0.128	0.004	0.127	29.536***	0.133	0.004	0.132	29.860***	0.130	0.005	0.125	27.953***
Physical activity			0.184	0.004	0.262	46.170***	0.172	0.004	0.257	46.930***	0.193	0.004	0.272	47.767***
Sedentary time for study purposes			-0.007	0.001	-0.028	6.439***	-0.008	0.001	-0.027	5.998***	-0.007	0.001	-0.024	5.420***
Sedentary time for purposes other than study			-0.017	0.001	-0.051	12.730***	-0.012	0.001	-0.041	9.899***	-0.014	0.001	-0.044	10.432***
Age			-0.020	0.002	-0.039	9.024***	-0.004	0.002	-0.008	1.726 ^*ns*^	0.006	0.002	0.012	2.806*
Gender			-0.101	0.009	-0.056	11.792***	-0.056	0.008	-0.031	6.580***	-0.027	0.009	-0.015	3.055*
Academic achievement			0.043	0.003	0.056	13.013***	0.038	0.003	0.049	10.930***	0.049	0.004	0.064	14.069***

Education of parents was associated with adolescents’ health only through health-related behaviors whereas the economic status of the family was associated with adolescents’ health both directly and through health-related behaviors. The standardized total effects between the education of parents and perceived health were -.003 in 2019, -.015 in 2020, and -.014 in 2021. The standardized total effects between the economic status and perceived health were .149 in 2019, .149 in 2020, and .142 in 2021.

Education of parents was more related with sedentary time, especially sedentary time for study purposes, than physical activities, compared with the economic status of the family. In contrast, the economic status of the family was more related with physical activities than sedentary time, compared with education of parents. Physical activities are positively associated with perceived health, but sedentary time was negatively related to perceived health regardless of the purposes of sedentary time.

There were similarities and differences in the association between variables across the years. Overall associations between socio-economic characteristics (education of parents, economic status of family) and sedentary time were stronger after Covid-19. Even though the association between parental education and sedentary time for study purposes marginally decreased immediately after Covid-19 (β in 2019 = .118, β in 2020 = .113), it increased in 2021 (β = .132). Education of parents was more positively associated with sedentary time for study purposes in 2021 than 2019, whereas it was more negatively associated with sedentary time for purposes other than study in 2021 than 2019. Economic status of the family was more associated with sedentary time for study purposes after the outbreak of Covid-19 (β in 2019 = .017, β in 2020 = .038, β in 2021 = .035).

## Discussion

Identifying changes in health-related behaviors and the perceived health of adolescents following the outbreak of Covid-19 is crucial for understanding adolescents’ health in this pandemic situation. Mechanisms between the socio-economic characteristics and outcomes (e.g., physical health, sedentary behavior, perceived health) using representative data would help to design effective public health interventions for adolescents. The main results showed that physical activity and sedentary time for study purposes reduced after the outbreak of Covid-19, whereas sedentary time for purposes other than study increased. Pathways to demographic characteristics and perceived health showed that socio-economic status was associated with adolescents’ perceived health through physical activity and sedentary time, but the association varied depending on dimensions of socio-economic characteristics and types of sedentary time.

Adolescents’ physical activity declined immediately after Covid-19 (2020). The results are consistent with previous studies that reported a decline in physical activity during Covid-19 [3, 19]. It is important to note that there was no difference in physical activity levels in 2019 and 2021. Evidence around longer-term trajectory indicates that there has been a recovery in physical activity among adolescents. These results may be associated with reduced rates of school closure in 2021. In 2020, school closure rates were 53.7% for middle school and 45.2% for high school. However, the ratios decreased in 2021 (36.2% for middle school and 28.0% for high school) [20]. Participating in physical activities in school and recognizing its importance during Covid-19 would help to recover the level of physical activity in 2021. This explanation should be evaluated in further studies.

Adolescents’ sedentary time for studying purposes was lowest in 2020 and remained lower in 2021 than in 2019. However, the sedentary time for purposes other than studies was highest in 2020 and rose further in 2021 than in 2019. This result indicates that categorizing sedentary time based on the study purpose is crucial to understand changes in adolescents’ sedentary behaviors. Previous studies have found that sedentary behavior significantly increased after Covid-19 [19]. Based on the results of the current study, we can assume that the increased sedentary time is not for studying, but for recreational screen time [21]. The result is also in line with previous studies demonstrating that sitting for leisure activities such as video games, TV viewing, and browsing the internet accounted for 84% of sedentary behaviors in children (ages 5 to 13) [22]. Since sedentary time for purposes other than study was negatively associated with perceived health, public programs for reducing recreational sedentary time would be effective to promote adolescents’ health.

Unlike the results of health-related behaviors, adolescents’ perceived health was lowest in 2021. This result suggests that the negative effects of Covid-19 on students’ subjective health are ongoing. The current study found the reason for decreased perceived health by investigating pathways among socio-economic characteristics and health-related behaviors. Since the health compromising active lifestyle was worsening in 2020 (i.e., lowest physical activity, highest sedentary time not for studying purposes), the delayed effects would emerge in 2021. Studies tracking longitudinal changes of perceived health and influencing factors are needed.

Adolescents’ socio-economic status was associated with perceived health through health-related behaviors. These indicate that intervention in health-related behavior would be an effective way to solve health inequity. However, overall extents of coefficients were not large. The absolute value of standardized regression coefficients between socio-economic characteristics and health-related behaviors ranged from 0.02 to .13. It is consistent with the theoretical explanation of modernity that socio-economic status is less important to explain characteristics in adolescence [23]. In the same vein, the association between socio-economic status and health is clear in adults, while it becomes vague in adolescents, which is one of the most critical periods that change their future. Given that even a small change during adolescence could cause a significant difference in adulthood, a detailed understanding of each influencing variable is fundamental.

Education of parents and economic status of the family is associated with adolescents’ health through health-related behaviors. These results support the FCT that resources related to health are determined by socio-economic status [24]. FCT claims multiplicity pathways between socio-economic status and health and the results of the current study showed that physical activity and sedentary behavior would be possible ways. Especially, parental education showed full mediation. Parental education has an impact on adolescents’ only through health-related behaviors [6]. Intervention for developing healthy habits among adolescents would help reduce health disparities in educational status.

The association between socio-economic status and health-related behaviors is complex. Having parents with higher levels of education reduces adolescents’ physical activity, but high income of family increases adolescents’ physical activity. The directions of socio-economic status varied depending on the domain of sedentary time. High socio-economic status increases sedentary time for study purposes and decreases sedentary time for purposes other than study. Understanding these complicated associations would disentangle inconsistent results of previous studies. Distinguishing education, economic status, and categorizing sedentary time by purposes, not considering them as one component, would help understand the association between socio-economic status and health.

Adolescents whose parents had a high level of education were less active, suggesting that the education of parents would be a risk factor for adolescents’ health [25]. Compared with high school-educated mothers, adolescents with university-educated mothers have more sedentary lifestyles and are less active [13]. However, opposite association has been reported in children [26]. Compared to children (ages 6 to 9) with low parental education, the likelihood of lower participation in physical activity decreased in children with high parental education [26]. It can be interpreted that highly educated parents emphasize the importance of studying more than physical activity as their child grows. These interpretations should be empirically investigated.

Economic status of the family is positively associated with physical activity and sedentary time for study purposes, negatively associated with sedentary time for purposes other than study. The results are consistent with previous studies finding that higher income was related to lesser screen time [27], which can treat as a proxy of recreational sedentary time. Lack of practicing sports was significant in children from low income families [26]. Further studies are needed to comprehensively determine the association between income and high physical activity in adolescents. In addition, it is worthy to note that the association is different in adult samples. There is a positive association between monthly personal income and sedentary behavior in adults [28]. This is because adults with lower income tend to be employed in a physically active job compared with adults with high income who are employed in electronic/computer-based jobs. Therefore, considering participants’ age is critical for understanding economic status and health-related behaviors.

Physical activity is positively associated with perceived health, but sedentary time is negatively related to perceived health regardless of the purposes of sedentary time. The coefficients with perceived health were larger between physical activity and perceived health (β = .26 to .27) than between sedentary behaviors and perceived health (β = -.02 to -.04). Physical activity was a critical predicting factor for depression and anxiety during the pandemic [29]. Public policy and educational interventions would be geared to be encouraging, especially for physical activity and reducing sedentary behaviors is critical to ameliorate problems during the pandemic.

Overall associations between socio-economic characteristics (education of parents, economic status of family) and sedentary time appeared stronger after the outbreak of Covid-19. Even though the association between the education of parents and sedentary time for study purposes marginally decreased immediately after Covid-19 (β in 2019 = .118, β in 2020 = .113), it increased in 2021 (β = .132). There are limited studies that directly demonstrated the differences of association before and after Covid-19, which hiders the theoretical explanation, but it is plausible that these results could be attributed to restricted public education and increased staying time at home during Covid-19. However, since the difference is not stable, further studies and evidence is needed.

There are several limitations in this study. First, the survey instrument utilized was not under the researcher’s control. Although the measurement was developed and validated by researchers of KDCA institute, there may be another validated questionnaire available, such as the International Physical Activity Questionnaire (IPAQ) to measure physical activity. Further studies are needed to determine if the same results can be obtained using other measures. Second, the current study used covariance-based structural equation modeling (SEM) to examine the associations among the variables. Covariance-based SEM is used because it is more suitable to verify a theoretical model with directional arrows representing hypothesized causal relations among the latent variables. However, partial least squares (PLS) SEM can also be used to explore associations among the variables or predictive tasks. Therefore, further studies are needed to explore other statistical approaches such as PLS-SEM. Third, adolescents’ demographic characteristics, including age, gender, and academic achievement, were entered as control variables. However, there may be critical variables related to health and health behaviors, such as obesity, sleep time, or diet. Further studies should consider these variables to understand the associations between socio-economic characteristics and health.

## Conclusion

Adolescence is a critical time to inculcate healthy habits that persist into adult life. Global concerns about adolescents’ unhealthy behaviors such as lower physically activity and sedentary lifestyles have been continued and highlighted especially in the wake of Covid-19. Education of parents and economic status of the family could play both a negative and positive role on adolescents’ health by reducing or increasing physical activity and sedentary time for studying purpose. Health-related behaviors can be potential targets of strategies to reduce socio-economic disparities and promote adolescents’ health during Covid-19.
